# Lesion-to-background ratio threshold value of SUVmax of simultaneous [^68^Ga]Ga-PSMA-11 PET/MRI imaging in patients with prostate cancer

**DOI:** 10.1186/s13244-020-00926-y

**Published:** 2020-12-17

**Authors:** Jing Zhao, Bernd Hamm, Winfried Brenner, Marcus R. Makowski

**Affiliations:** 1 Institute of Radiology and Nuclear Medicine, Charité – Universitätsmedizin Berlin, corporate member of Freie Universität Berlin, Humboldt-Universität zu Berlin, and Berlin Institute of Health, Charitéplatz 1, 10117 Berlin, Germany; 2 Institute of Nuclear Medicine, Charité – Universitätsmedizin Berlin, corporate member of Freie Universität Berlin, Humboldt-Universität zu Berlin, and Berlin Institute of Health, Augustenburger Platz 1, 13353 Berlin, Germany; 3 Institute of Diagnostic and Interventional Radiology, Klinikum Rechts Der Isar, Technische Universität München, Ismaninger Str. 22, 81675 Munich, Germany

**Keywords:** Prostate cancer, Multiparametric MRI, PSMA, [^68^Ga]Ga-PSMA PET/MRI, SUVmax

## Abstract

**Purpose:**

This study aimed to calculate an applicable relative ratio threshold value instead of the absolute threshold value for simultaneous ^68^Ga prostate-specific membrane antigen/positron emission tomography ([^68^Ga]Ga-PSMA-11 PET) in patients with prostate cancer (PCa).

**Materials and methods:**

Our study evaluated thirty-two patients and 170 focal prostate lesions. Lesions are classified into groups according to Prostate Imaging Reporting and Data System (PI-RADS). Standardized uptake values maximum (SUVmax), corresponding lesion-to-background ratios (LBRs) of SUVmax, and LBR distributions of each group were measured based on regions of interest (ROI). We examined LBR with receiver operating characteristic analysis to determine threshold values for differentiation between multiparametric magnetic resonance imaging (mpMRI)-positive and mpMRI-negative lesions.

**Results:**

We analyzed a total of 170 focal prostate lesions. Lesions number of PI-RADS 2 to 5 was 70, 16, 46, and 38. LBR of SUVmax of each PI-RADS scores was 1.5 (0.9, 2.4), 2.5 (1.6, 3.4), 3.7 (2.6, 4.8), and 6.7 (3.5, 12.7). Based on an optimal threshold ratio of 2.5 to be exceeded, lesions could be classified into MRI-positive lesion on [^68^Ga]Ga-PSMA PET with a sensitivity of 85.2%, a specificity of 72.0%, with the corresponding area under the receiver operating characteristic curve (AUC) of 0.83, *p* < 0.001. This value matches the imaging findings better.

**Conclusion:**

The ratio threshold value of SUVmax, LBR, has improved clinical and research applicability compared with the absolute value of SUVmax. A higher threshold value than the background’s uptake can dovetail the imaging findings on MRI better. It reduces the bias from using absolute background uptake value as the threshold value.

## Key points

- The ratio threshold value of SUVmax, LBR, has improved clinical and research applicability compared with the absolute value of SUVmax/
- A higher threshold value than the background’s uptake can dovetail the imaging findings on MRI better.
- The specificity of [^68^Ga]Ga-PSMA PET needs to be further improved.

## Background

Prostate cancer (PCa) is a common malignant disease in the elderly male population. Approximately 17% of patients with early prostate cancer have metastatic disease. PCa is the second leading cause of cancer death in men in the western world [1].

Multiparametric magnetic resonance imaging (mpMRI) has been a clinical imaging tool for detecting primary PCa and guiding subsequent biopsy. MpMRI includes T2-weighted imaging (T2WI), diffusion-weighted imaging (DWI), apparent diffusion coefficient (ADC), and dynamic contrast-enhanced MRI (DCE-MRI). Prostate Imaging Reporting and Data System (PI-RADS) interprets results [2, 3].

PSMA is a transmembrane glycoprotein related to tumor progression and disease recurrence. PSMA over-expresses in prostate cancer cells. It is associated with PCa with higher serum prostate-specific antigen (PSA) levels and a higher Gleason score (GS) [4, 5].

Positron emission tomography (PET) images are co-registered with computed tomography (CT) scans. CT is easily acquired and widely available to provide anatomical information about the localization of PSMA-avid lesions. Previous studies suggest that [^68^Ga]Ga-PSMA-11 PET/CT has a high detection rate for prostate tumors, with a sensitivity of 67–97% [6, 7]. Koerber et al. [8] and Woythal et al. [7]reported that SUVmax of PCa is higher than that of non-cancerous lesions and healthy prostate tissue. Combining [^68^Ga]Ga-PSMA-11 PET and mpMRI has the potential to improve localization accuracy and diagnostic efficiency, as Zamboglou et al. proved [9]. In both studies, experts elaborated on the advantages of PET/MRI in the diagnosis of PCa.

Nevertheless, two aspects can be further optimized. First, MRI-positive lesions may show unapparent or low uptake in PET images. MRI-negative lesions may show apparent uptake in PET images. It may misdiagnose part of MRI-negative lesions as positive if we consider all apparent uptake lesions as positive in PET images. Therefore, it is necessary to increase the threshold value, which is higher than the background SUVmax.

Second, in most publications, individual research centers adopt its threshold standard to proceed with studies. The threshold standard varies from different medical centers. Hence, each study is conducted under different execution standards. Eiber et al. [10] took SUVmax higher than the background as a threshold value to prove diagnostic accuracy improvement. Woythal et al. [7]reported the best threshold value of 3.15 with sensitivity 97%, specificity 90%, and area under curve (AUC) 0.987. Donato et al. [11] described lesions as mildly avid (SUVmax < 5), moderately avid (SUVmax > 5), or intensely avid (SUVmax > 10). Hicks et al. [12]calculated a threshold of 6.7, with sensitivity 88%; specificity 96%.

However, SUVmax is affected by a specific combination of radiotracer manufacturer, systems vendor, reconstruction techniques, uptake time, post-processing software, the time between radiotracer injection and scanning, and even the human race. Taking absolute value for research results in bias from different imaging conditions. Therefore, in our study, we used ratio value LBR to perform research.

We aimed to classify prostate lesions according to MRI morphological imaging analysis to achieve a better threshold LBR value. This LBR threshold value matches the imaging findings on MRI better. It reduces the possibility of MRI-negative lesions being misdiagnosed as positive in PET images. It reduces the bias from using absolute background uptake value as the threshold value. We also re-examined clinical follow-up information and subsequent pelvic MRI to verify whether the lesion is radiological positive or negative.

## Materials and methods

### Study population

This retrospective study was approved by the institutional ethics review board (EA1/060/16), and the institutional review board waived the requirement for informed consent for this retrospective analysis.

Inclusion criteria are as follows: (1) patients with biopsy-proven PCa who underwent simultaneous [^68^Ga]Ga-PSMA-11 PET/MRI between January 2017 and March 2020 in our department; (2) all necessary additional information could be obtained from clinical records; (3) patients underwent pelvic MRI examination at our institution for follow-up analysis. Exclusion criteria are as follows: (1) patients who underwent prostatectomy before scanning; (2) patients whose follow-up information is not adequate.

### [^68^Ga]Ga-PSMA-11 PET/MRI imaging protocol

[^68^Ga]Ga-PSMA-11 was synthesized using a clinical-grade ^68^Ge/^68^ Ga radionuclide generator (Eckert & Ziegler Radiopharma GmbH, Berlin, Germany) and PSMA-HBED-CC (ABX GmbH, Radeberg, Germany) as described previously [13–15]. Patients were imaged after 83 ± 12 min after intravenous injection of a mean activity of 161.0 ± 21.4 MBq (4.4 ± 0.6 mCi) [^68^Ga]Ga-PSMA-11, activity: 1.8–2.2 MBq (0.049–0.060 mCi) per kilogram bodyweight. No adverse effects were observed after the injection of [^68^Ga]Ga-PSMA-11. Furosemide is injected to minimize halo artifact caused by scatter overcorrection associated with high renal and urinary tracer activity 0.5 h before the scan. Patients void urine right before the start of the examination.

Imaging was performed with a 3.0 T PET/MRI system (SIEMENS MAGNETOM Biograph mMR, Erlangen, Germany). Every patient uses the same protocol of PET and MRI scanning. The acquisition contains two parts. First, body PET/MRI from the vertex to mid-thigh was performed with 3 min of PET acquisition in each bed position, each 24 cm. Two six-element body matrix coils placed anteriorly were used in conjunction with two posterior spine clusters to optimize the signal-to-noise ratio (SNR) in the MRI scanner. A Dixon 3D volumetric interpolated breath-hold examination (VIBE) T1-weighted MRI sequence was performed at each bed position and used for the generation of attenuation maps and anatomic allocation of the PET results. Siemens StarVIBE overcomes motion artifacts.

The second part was a dedicated MRI scan of the pelvis, followed by the reconstruction of PET data. Reconstruction was conducted with an ordered subset expectation maximization algorithm (OSEM), with 3 iterations/21 subsets, based on an x-matrix acquisition with a 4-mm Gaussian filter and relative scatter scaling. Attenuation correction was performed using the non-enhanced MRI data. Table 1 summarizes MRI imaging parameters.

**Table 1:** Imaging parameters used for MRI

Sequence	TR/TE(msec)	FOV(mm)	Flip angle (°)	Section thickness (mm)	Voxel size (mm)
T2WI HASTE Axial	1400.0/95.0	400	160	5.0	1.3 × 1.3 × 5.0
T1WI FS VIBE	1600.0/96.0	350	160	4.0	1.1 × 1.1 × 4.0
T2WI Axial	5500.0/103.0	180	150	3.0	0.5 × 0.5 × 3.0
T2WI Sagittal	1600.0/96.0	350	160	4.0	1.1 × 1.1 × 4.0
T2WI Coronal	4500.0/102.0	200	173	3.0	0.4 × 0.4 × 3.0
DWI	11,600.0/70.0	280		3.0	2.5 × 2.5 × 3.0
T1WI FS TWIST dynamic	7.41/3.30	260	12	3.5	1.4 × 1.4 × 3.5
T1WI STARVIBE	3.71/1.77	360	9	1.2	1.1 × 1.1 × 1.2

### Image analysis

Image analysis was performed on a Visage 7.1 Workstation (Visage Imaging GmbH, Berlin, Germany). All mpMRI images were interpreted by a board-certified radiologist with more than fifteen years without access to the PET images, following the PI-RADS criteria, version 2 [16]. The readers classified prostate focal lesions with PI-RADS scores of 2 and 3 as MRI negative, while 4 and 5 as MRI positive. The present analysis excluded PI-RADS 1 because we do not report PI-RADS 1 lesions. T2WI was used for anatomic correlation for [^68^Ga]Ga-PSMA-11 PET.[^68^Ga]Ga-PSMA-11 PET scans were read by a nuclear medicine specialist with more than ten years of experience, who was not aware of the MRI results. ROI was defined as a region with an abnormal signal in MRI images or avid PSMA uptake in PET images. SUVmax is measured based on ROI. Any avid focal lesion in the prostate with uptake above prostate background not attributable to physiologic radiotracer biodistribution was considered positive in [^68^Ga]Ga-PSMA-11 PET. Lesions with the same or lower uptake than background were considered negative in [^68^Ga]Ga-PSMA-11 PET. Besides, background SUVmax was measured in the nearest visually defined normal tissue adjacent to a lesion as background uptake 1.0cm^2^, a perfect circle. LBR is defined as a ratio of lesion SUVmax to background SUVmax. Readers resolved discrepancies based on a separate consensus reading. Both interpreters reviewed all imaging studies in a single session.

### Statistical analysis

We classified prostate lesions into four groups according to PI-RADS from 2 to 5 and calculated the LBR of each group. Additionally, we classified LBR into four levels, including LBR ≤ 1, 1 < LBR ≤ 2, 2 < LBR ≤ 3, LBR > 3, and analyzed how does LBR of each PI-RADS group distribute.

To estimate the optimal LBR threshold, we performed ROC analysis and calculation of the AUC. Youden’s index defined the optimal cutoff value. Youden’s index= sensitivity + specificity − 1. In order to present the threshold’s effect on sensitivity and specificity, we also calculated the sensitivity and specificity corresponding to the other six thresholds, besides the optimal threshold.

Two-sided *p *values < 0.05 were considered statistically significant. All statistical analyses were performed using SPSS 25 for Windows (IBM Corp, Armonk, NY). The significance level was set to *α* < 0.05. Patient demographics and clinical characteristics are summarized using descriptive statistics. Normally distributed data are reported as mean ± SD, and non-normally distributed data are reported as medians (interquartile range, IQR Q1, Q3).

## Result

### Characteristics of patients

**Fig. 1:**
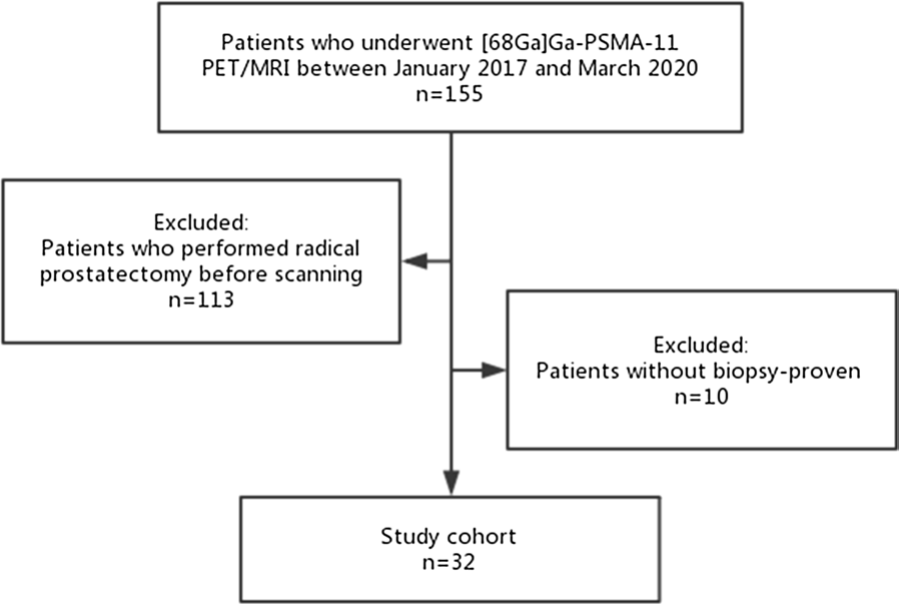
[^68^Ga]Ga-PSMA PET = gallium 68-labeled prostate-specific membrane antigen PET, mpMRI = multiparametric MRI

Thirty-two patients who underwent [^68^Ga]Ga-PSMA-11 PET/MRI without RP were retrospectively selected from the database and included for analysis. The PSA level of these patients was 11.45 (5.67–24.36) ng/mL. Figure 1 shows patients' inclusion and exclusion in the flowchart. Demographics are given in Table 2.

**Table 2:** Summary of patient characteristics

Parameter	Value
No. of patients	32
Age (yr.)	70 ± 7
PSA level (ng/mL)	11.45 (5.67, 24.36)
Clinical *T* stage	
T2a	2
T2b	2
T2c	3
T3a	8
T3b	8
T4	9
Biopsy GS	
6	4
7	
3 + 4	7
4 + 3	5
8	9
9	
4 + 5	2
5 + 4	3
10	2

### Corresponding LBR analysis

A total of 170 focal prostate lesions were detected. PI-RADS score was 2 in 70 lesions (70/170) with LBR of 1.5 (0.9, 2.4); 3 in 16 lesions (16/170) with LBR of 2.5 (1.6, 3.4); 4 in 46 lesions (46/170) with LBR of 3.7 (2.6, 4.8); and 5 in 38 lesions (38/170) with LBR of 6.7 (3.5, 12.7). LBR was classified into four levels, including LBR ≤ 1, 1 < LBR ≤ 2, 2 < LBR ≤ 3, and LBR > 3. Table 3 gives the distribution of each PI-RADS score group.

**Table 3:** LBR distribution of each PI-RADS score group

PI-RADS	LBR ≤ 1	1 < LBR ≤ 2	2 < LBR ≤ 3	LBR > 3
2	31	30	22	17
3	12	19	31	38
4	4	10	23	63
5	5	5	3	87

Data are described in percentage (%)

**Fig. 2:**
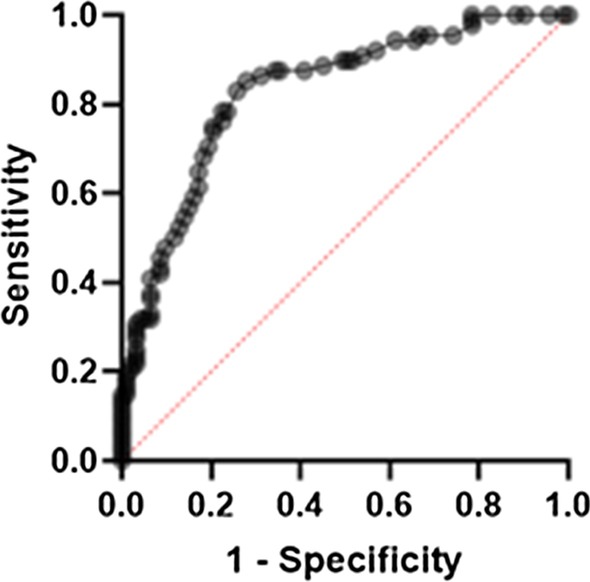
Receiver operating characteristic curves generated with a generalized linear model of LBR for [^68^Ga]Ga-PSMA PET. With the generalized linear model estimate, AUC for [^68^Ga]Ga-PSMA PET was 0.83, 95% CI (0.77, 0.89), 85.2% sensitivity, 72.0% specificity, *p* < 0.001

**Fig. 3:**
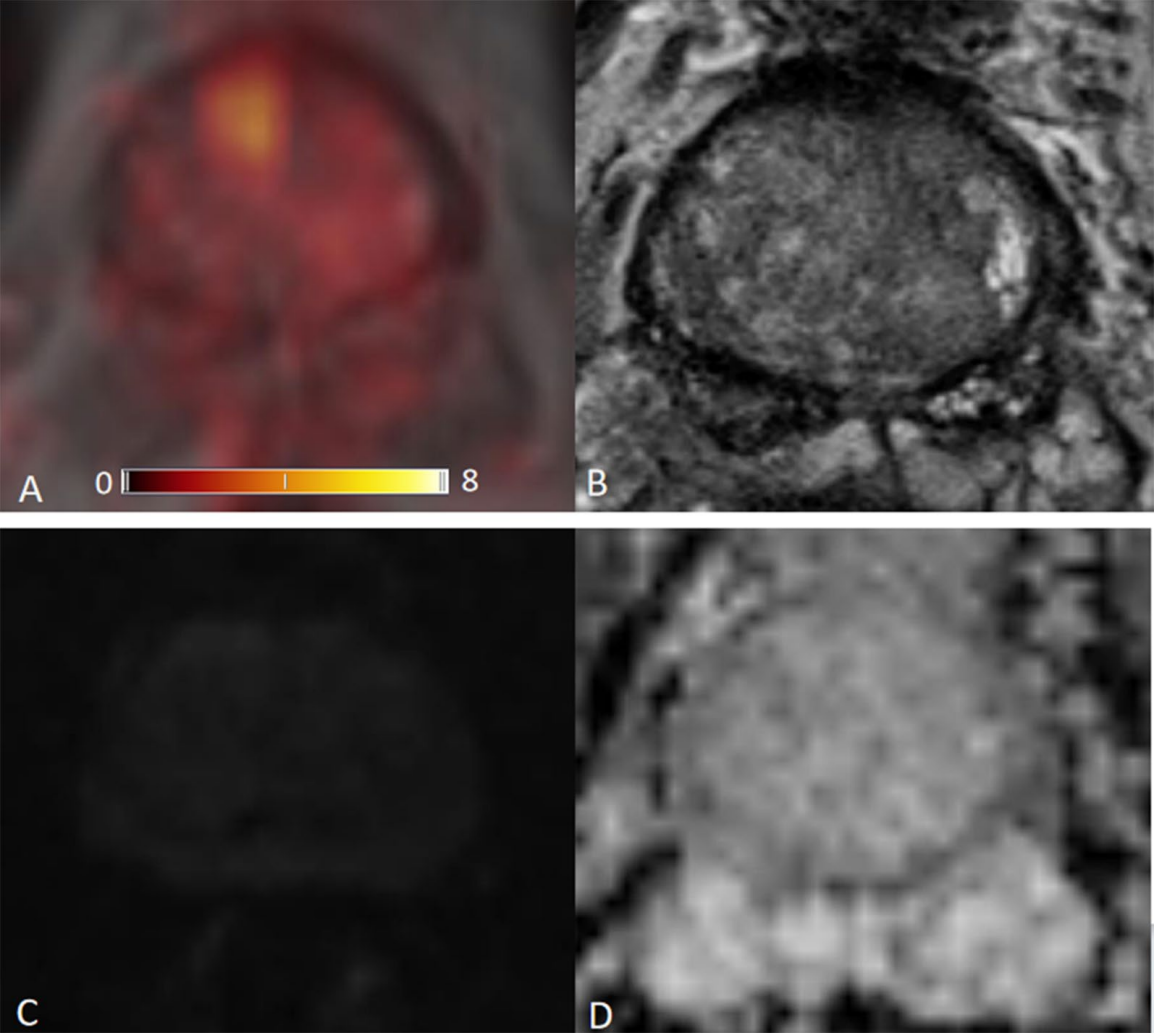
[^68^Ga]Ga-PSMA PET/MRI images obtained in a 63-year-old man, PSA 0.50 ng/mL. **a** [^68^Ga]Ga-PSMA PET/MRI; **b** T2WI; **c** DWI, *b* value 1000 s/mm^2^; **d** ADC. This example showed that normal prostate tissue and MRI-negative lesions show varying levels of PSMA uptake. The highest uptake in this figure is SUVmax 10.9, right anterior TZ, background SUVmax 1.1, LBR 9.9

**Fig. 4:**
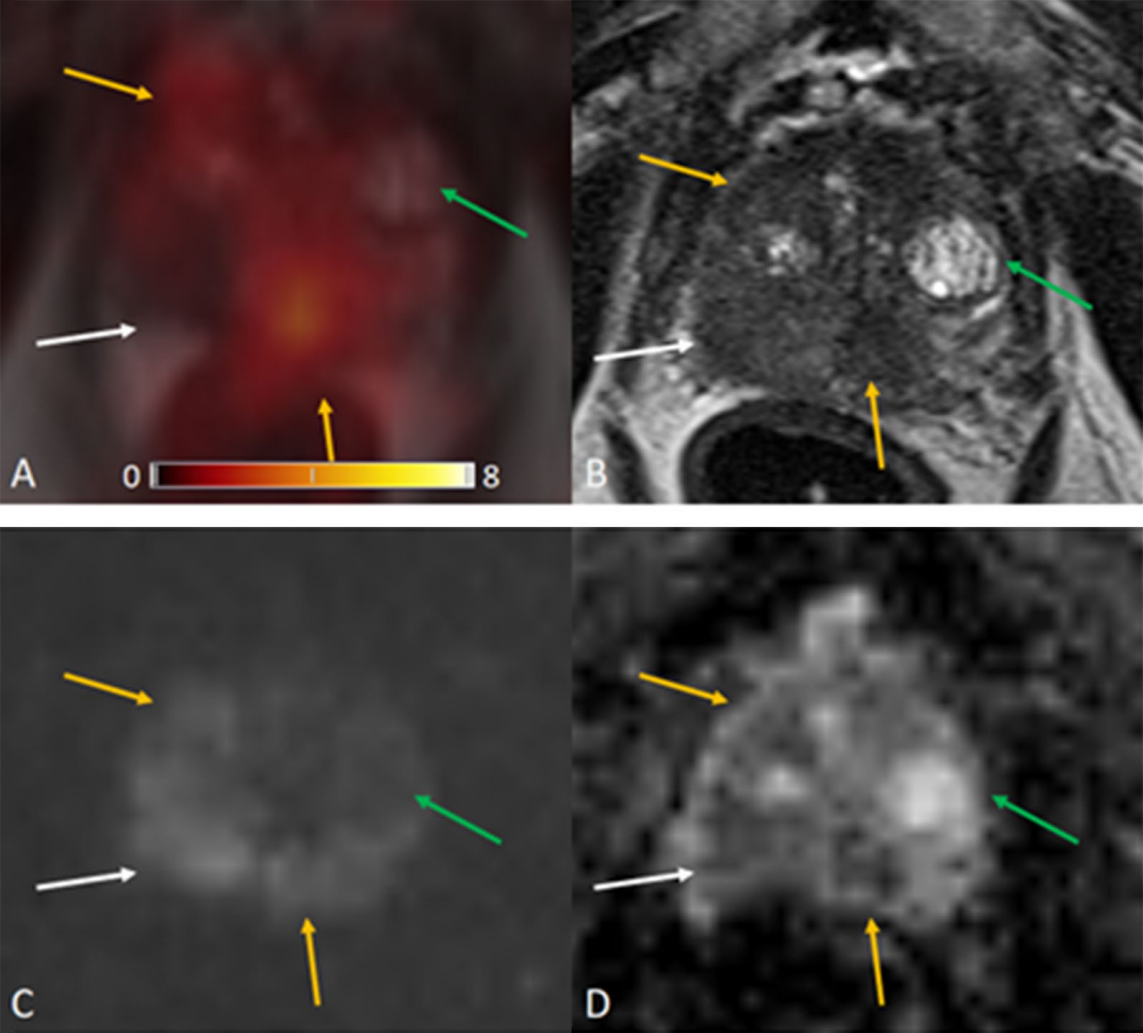
[^68^Ga]Ga-PSMA PET/MRI images obtained in a 79-year-old man, PSA 2.20 ng/mL. **a** [^68^Ga]Ga-PSMA PET/MRI; **b** T2WI; **c** DWI, *b* value 1000 s/mm^2^; **d** ADC. Background SUVmax 1.7. MRI-positive lesions are yellow arrows and white arrow. Yellow arrow: SUVmax 5.9, LBR 3.5 (middle of PZ) and 4.2, LBR 2.5 (right TZ). White arrow: SUVmax 1.6, LBR 0.9 (right PZ). MRI-negative lesion is green arrow, SUVmax 1.4, LBR 0.8 (left TZ). This example showed that MRI-positive lesions could be either PSMA avid uptake (yellow arrows) or unapparent PSMA uptake (white arrows). And MRI-negative lesions could be unapparent PSMA uptake (green arrows)

**Fig. 5:**
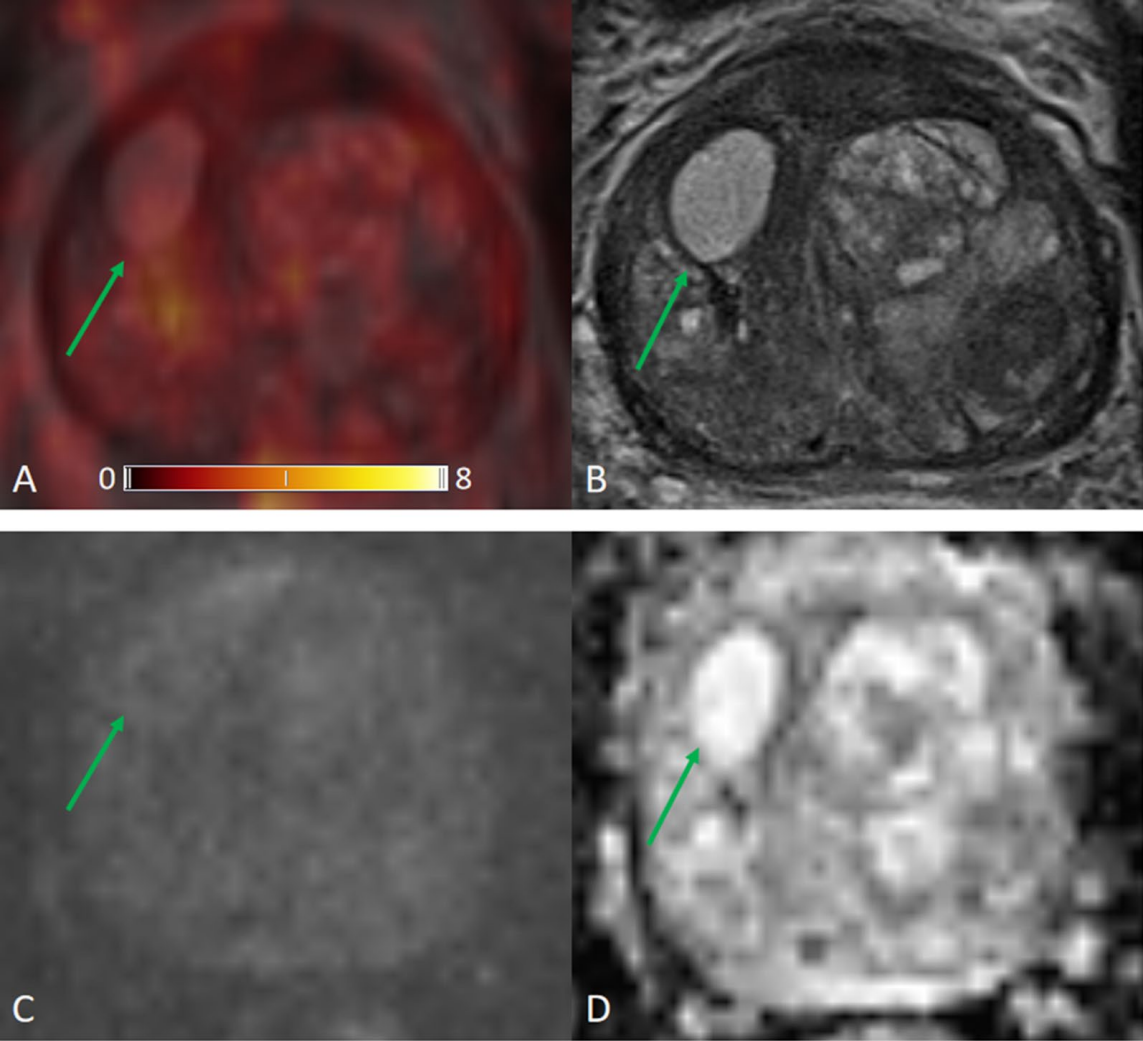
[^68^Ga]Ga-PSMA PET/MRI images obtained in a 72-year-old man, PSA 24.81 ng/mL. **a** [^68^Ga]Ga-PSMA PET/MRI; **b** T2WI; **c** DWI, *b* value 1000 s/mm^2^; **d** ADC. This example showed an MRI-negative lesions, a typical encapsulated nodule with unapparent radiotracer uptake in [^68^Ga]Ga-PSMA-11 PET. SUVmax 1.9, right TZ, background SUVmax 1.2, LBR 1.6

**Fig. 6:**
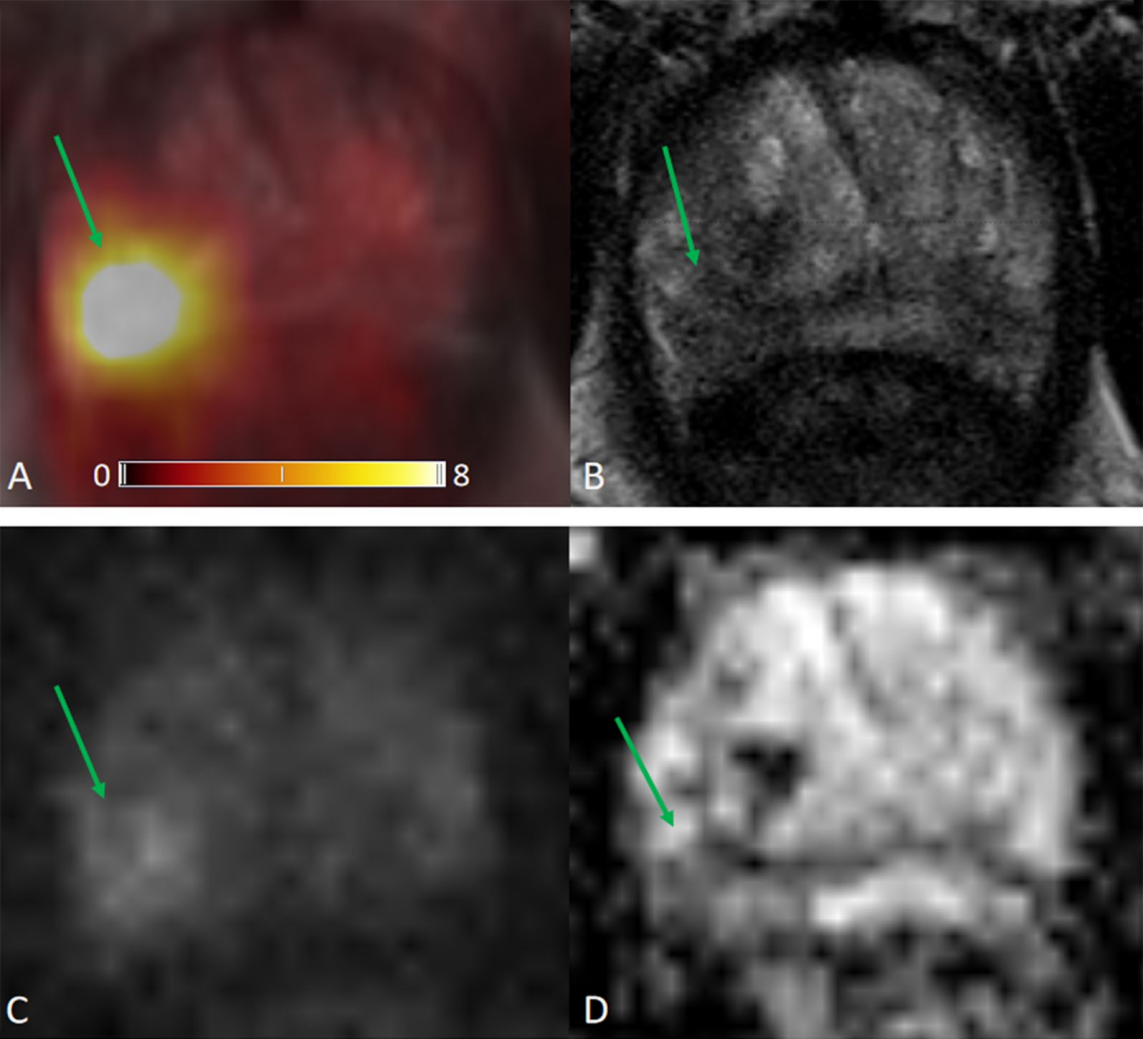
[^68^Ga]Ga-PSMA PET/MRI images obtained in a 76-year-old man, PSA 9.50 ng/mL. **a** [^68^Ga]Ga-PSMA PET/MRI; **b** T2WI; **c** DWI, *b* value 1000 s/mm^2^; **d** ADC. This example showed MRI-positive lesions, PI-RADS 4, with apparent PSMA uptake in [^68^Ga]Ga-PSMA-11 PET.a MRI-negative lesions, a typical encapsulated nodule with unapparent radiotracer uptake in [^68^Ga]Ga-PSMA-11 PET. SUVmax 13.6, right PZ, background SUVmax 1.5, LBR 9.1

The ROC for [^68^Ga]Ga-PSMA-11 PET and lesion validation results are shown in Fig. 2. The corresponding AUC for [^68^Ga]Ga-PSMA-11 PET was 0.83, 95% confidence interval (CI) (0.77, 0.89), with an optimal LBR threshold of 2.5 (85.2% sensitivity, 72.0% specificity), *p* < 0.001. Figure 3 provides an example of MRI-negative lesions and normal prostate tissue present varying levels of PSMA uptake in [^68^Ga]Ga-PSMA-11 PET. Figure 4 provides an example illustrating that MRI-positive lesions present apparent or unapparent radiotracer uptake in [^68^Ga]Ga-PSMA-11 PET. Figure 5 provides an example of MRI-negative lesions, a typical encapsulated nodule with unapparent radiotracer uptake in [^68^Ga]Ga-PSMA-11 PET. Figure 6 provides an example of MRI-positive lesions, PI-RADS 4, with apparent PSMA uptake in [^68^Ga]Ga-PSMA-11 PET.

We also calculated the sensitivity and specificity corresponding to the other six thresholds, besides the optimal threshold, to present the threshold’s effect on sensitivity, specificity, and Youden’s index, summarized in Table 4.

**Table 4:** Summary of sensitivity and specificity of thresholds

Threshold	Sensitivity	Specificity	Youden’s index
1.0	95.5%	31.2%	0.267
1.5	92.0%	43.0%	0.350
2.0	88.6%	54.8%	0.434
2.5	85.2%	72.0%	**0.572**
3.0	73.9%	79.6%	0.535
3.5	61.4%	82.8%	0.442
4.0	50.0%	88.2%	0.382

Bold value indicates the largest Youden’s index value

## Discussion

Our study shows that [^68^Ga]Ga-PSMA-11 PET presents high sensitivity of detecting prostate lesions. However, part of MRI-negative lesions show higher SUVmax than background SUVmax. It could lead to an over-diagnose of MRI-negative lesions and low specificity. A higher threshold value of [^68^Ga]Ga-PSMA-11 PET is needed instead of background uptake. To improve the clinical applicability of our study, we calculated lesion-to-background ratios, a relative ratio. In our study, the threshold LBR of 2.5 achieves a better clinical and research applicability to classify positive and negative lesions of [^68^Ga]Ga-PSMA-11 PET study.

We analyzed 32 patients with prostate cancer undergoing [^68^Ga]Ga-PSMA-11 PET/MRI. The accuracy of imaging examinations plays a crucial role in diagnosing prostate focal lesions. In our study, LBR revealed the comparison of lesion uptake and background uptake in PET images. LBR ≤ 1 means that lesion uptake is lower or equal to background uptake, as well as negative in [^68^Ga]Ga-PSMA-11 PET. LBR > 1 represents that lesion uptake is higher than background uptake, as well as positive on [^68^Ga]Ga-PSMA-11 PET. The higher the ratio, the greater the tendency of a lesion to be PET positive. In the prostate, increased expression of PSMA receptors is not characteristic only of prostate cancer cells, but may also occur in normal prostate cells or non-cancerous lesions such as benign prostatic hyperplasia (BPH), as shown in Fig. 3. Our [^68^Ga]Ga-PSMA-11 PET findings showed that MRI-negative focal lesions might also show a certain degree of PSMA uptake. While some of MRI-positive lesions showed unapparent or mild in PSMA PET, the majority MRI-positive lesions exhibited moderate to strong PSMA avidity, as shown in Fig. 4.

Our lesion-based LBR analysis shows that higher LBR of SUVmax tends to indicate a higher likelihood of malignancy. The higher the PI-RADS score lesions group, the more significant the proportion of LBR > 3. Hence, taking prostate background SUVmax as a threshold value to identify PET positive or negative is relatively low. Our results suggest that the use of prostate background’s SUVmax as a threshold value for differentiating MRI negative from MRI-positive prostate lesions can cause in a relevant number of false-positive cases. LBR is defined as a ratio of lesion SUVmax to background SUVmax. For metastasis, background SUVmax is the uptake value of nearby normal tissue, including normal bone tissue and normal soft tissue. These normal tissues usually do not show PSMA avid uptake. The background SUVmax of metastasis is relatively low. The difference between lesions and background is more pronounced. Therefore, the optimal LBR threshold of 2.5 can also be used for metastasis.

Both mpMRI and [^68^Ga]Ga-PSMA-11 PET/CT have been widely used imaging techniques in detecting prostate cancer. Earlier studies have revealed the usefulness of [^68^Ga]Ga-PSMA-11 PET to detect prostate lesions patients. Hope et al. performed a meta-analysis of [^68^ Ga]Ga-PSMA-11 PET accuracy for the detection of PCa and demonstrated a sensitivity and specificity of 0.74 [17]. Hirmas et al. reported high performance for the detection of lymph node metastasis and bone metastasis. It revealed a significantly higher concordance rate of 90%, compared to the bone scan of 75%, MRI of 73%, and CT of 60% [18]. The benefit of it is a comprehensive scanning range and high sensitivity, and [^68^Ga]Ga-PSMA-11 PET is widely used to achieve accurate staging and post-treatment efficacy evaluation. Therefore, more extensive use of [^68^Ga]Ga-PSMA-11 PET shortens the time of prostate metastatic lesion detection and improves clinical decision-making.

MRI brings valuable superiority over [^68^Ga]Ga-PSMA-11 PET/CT because of the high soft-tissue contrast and provides the advantages of functional MRI techniques, as Hoeks et al. [19] demonstrated. Some attempts have been made to provide a multimodality approach. Park et al. [20] found that [^68^Ga]Ga-PSMA-11 PET can be used to identify prostate cancer, while MR imaging provides detailed anatomic guidance. Therefore, [^68^Ga]Ga-PSMA-11 PET/MRI imaging provides valuable diagnostic information and may inform the need for and extent of pelvic node dissection. Domachevsky et al. [21] proved that pelvic [^68^Ga]Ga-PSMA-11 PET/MRI is superior to whole-body [^68^Ga]Ga-PSMA-11 PET/CT in detecting extensions of localized disease. It is mainly due to the high soft-tissue resolution of MRI, by comparing between pelvic [^68^Ga]Ga-PSMA-11 PET/MRI and whole-body [^68^Ga]Ga-PSMA-11 PET/CT for the initial evaluation of prostate cancer. Abd-Alazeez et al. [22] studied the added value of apparent diffusion coefficient maps and dynamic contrast-enhanced images for the detection of radio recurrent prostate cancer and proved that MRI could evaluate recurrent or residual disease.[^68^Ga]Ga-PSMA-11 PET/MRI has also been used to detected metastasis. Kranzbühler et al. [23] reported the usage of [^68^Ga]Ga-PSMA-11 PET/MRI-positive peritoneal metastasis in the falciform ligament in recurrent prostate cancer. In conclusion, the development of MRI technology has dramatically improved the diagnostic accuracy of prostate cancer.

Nevertheless, PI-RADS is not perfect yet. Westphalen et al. critically evaluated the PI-RADS interpretation in 26 centers and reported that the positive predictive value of PI-RADS varied widely across centers [24]. The reason is that the efficacy of PI-RADS is generally related to the personal experience of physicians in practical application. Urologists and radiologists are still working on further optimizing the scoring system. Therefore, it is necessary to use a multimodality quantitative analysis to provide more information on diagnosis. For the interpretation of PET images, a five-point ordinal scale, Likert-scale can be utilized with a score of 1, meaning PCa was highly unlikely and a score of 5, meaning PCa was highly likely. For interpretation of PET and PET/MRI images in PCa lesions, we may consider LBR lower than 1 as highly unlikely, LBR between 1 and 2 as unlikely, respectively, LBR between 2 and 3 as equivocal, LBR higher than 3 as likely, respectively, and LBR higher than 4 as highly likely.

Afshar-Oromieh et al. and Guberina et al. have proved [^68^Ga]Ga-PSMA-11 PET/MRI could be the ideal imaging modality for staging PCa and clarify unclear findings on PET/CT [25, 26]. Uslu-Besli et al. demonstrated that SUV and ADC values are inversely correlated in primary prostate lesions. They combined both values' usage to increase the diagnostic accuracy of hybrid PET/MRI in the detection of primary prostate lesions and lymph node metastasis [27]. Park et al. studied patients with intermediate- or high-risk cancer. They proved that [^68^Ga]Ga-PSMA-11 can be used to identify prostate cancer, while MR imaging provides detailed anatomic guidance [20]. In terms of tumor severity and evaluation of extracapsular and seminal vesicular invasion, the results of [^68^Ga]Ga-PSMA-11 PET were encouraging. These parameters are significant considerations in treatment planning. If none of these findings exist, surgery can be performed. von Klot et al. studied that men who retain extracapsular extension may not undergo nerve-sparing surgical techniques. It leads to an increased risk of urinary incontinence and erectile dysfunction after prostatectomy [28]. These factors also have a profound impact on prognosis because both extracapsular extension and seminal vesicle invasion are associated with an increased risk of recurrence and lymph node and bone metastasis.

Because of the high sensitivity of PSMA, it is easier to detect hidden residual and recurrent focals [5, 22, 23, 25, 26, 29–32]. [^68^Ga]Ga-PSMA PET was superior to MRI in determining distant metastasis in patients with moderate- to high-risk PCa. As Roach et al. and Calais et al. demonstrated, this method becomes more widely used in clinical settings. Many patients with N0 or M0 staging, as assessed by current imaging, will more accurately stage N1 or M1 [33, 34]. The success of conventional imaging staging depends on whether the scanning range can fully cover the relevant parts. Preconditioning staging of [^68^Ga]Ga-PSMA-11 PSMA PET may be established as it scans the whole body.

However, [^68^Ga]Ga-PSMA-11 PET/MRI still has some drawbacks to overcome. First, hybrid PET/MRI is high-cost equipment. Many medical centers are not able to perform PET/MRI scanning before patients had RP. [^68^Ga]Ga-PSMA-11 PET/CT is relatively affordable equipment for medical centers and an affordable examination for patients, compared to PET/MRI. Doctors take [^68^Ga]Ga-PSMA-11 PET/CT as a regular examination for primary staging before performing radical prostatectomy. In this condition, there are more studies on [^68^Ga]Ga-PSMA-11 PET/CT. These researches can take radical prostatectomy specimens as the reference standard to perform lesion by lesion study. Chen et al. retrospectively enrolled patients who underwent both MRI and PET/CT before radical prostatectomy and analyzed the molecular imaging PSMA expression score and the pathologic results [35].

Second, [^68^Ga]Ga-PSMA PET/MRI needs a more extended scanning protocol than [^68^Ga]Ga-PSMA-11 PET/CT. During the scanning process, the MRI device emits a harsh noise. Although technicians adopt sound insulation solutions to patients, they still cannot eliminate the interference from noise to patients. The patient needs to keep the body stable and immobile during the entire scan.

Third, although [^68^Ga]Ga-PSMA-11 has been one of the milestone discovery in the development of nuclear medicine in recent decades, which significantly improves the accuracy of prostate cancer diagnosis and assessment. Its specificity still could be further enhanced. Optimizing the targeting specificity of molecular probes is one of the most important methods. We hope that this problem will be solved in the future.

In the end, [^68^Ga]Ga-PSMA-11 PET/MRI is widely used for staging reevaluation with recurrent prostate cancer after radical prostatectomy or to evaluate the conditions of patients who have already been treated by non-surgical therapies. Radical prostatectomy and prostate biopsies are invasive procedures with a high risk of focal hemorrhages and infection. PCa patients are mostly elderly men, with some underlying disease or age-related diseases that are not recommended to perform a pathological examination under this situation. To some extent, histopathological examination is not often feasible due to ethical and practical reasons.

### Limitations

The limitation of our retrospective analysis is that [^68^Ga]Ga-PSMA-11 PET/MRI is not compared with full histopathology examination because our cohort patients are elderly male and were not feasible to perform RP. Therefore, this analysis is a descriptive radiological imaging features study.

## Conclusion

The ratio threshold value of SUVmax, LBR, has improved clinical and research applicability compared with the absolute value of SUVmax. A higher threshold value than the background’s uptake is capable of dovetailing the imaging findings on MRI better. The specificity of [^68^Ga]Ga-PSMA PET needs to be further improved by optimizing the targeting specificity of molecular probes.

## Data Availability

The datasets analyzed and generated during this study are included in this published study.

## Abbreviations

ADC: Apparent diffusion coefficient
CI: Confidence interval
CT: Computed tomography
DCE-MRI: Dynamic contrast-enhanced MRI
DWI: Diffusion-weighted imaging
FOV: Field-of-view
Ga: Gallium
GS: Gleason score
IQR: Interquartile range
mpMRI: Multiparametric magnetic resonance imaging
MRI: Magnetic resonance imaging
OSEM: Ordered subset expectation maximization algorithm
PCa: Prostate cancer
PET/CT: Positron emission tomography/computed tomography
PET/MR: Positron emission tomography/magnetic resonance
PI-RADS: Prostate Imaging Reporting and Data System
PROMISE: Prostate Cancer Molecular Imaging Standardized Evaluation
PSA: Prostate-specific antigen
PSMA: Prostate-specific membrane antigen
ROI: Region of interest
SUV: Standardized uptake value
T2WI: T2-weighted imaging
TE: Echo time
TR: Repetition time

## References

[1] Siegel RL, Miller KD, Fedewa SA (2017). Colorectal cancer statistics. CA Cancer J Clin.

[2] Muller BG, Shih JH, Sankineni S (2015). Prostate cancer: interobserver agreement and accuracy with the revised prostate imaging reporting and data system at multiparametric MR imaging. Radiology.

[3] Rosenkrantz AB, Ginocchio LA, Cornfeld D (2016). Interobserver reproducibility of the PI-RADS version 2 lexicon: a multicenter study of six experienced prostate radiologists. Radiology.

[4] Ross JS, Sheehan CE, Fisher HAG (2003). Correlation of primary tumor prostate-specific membrane antigen expression with disease recurrence in prostate cancer. Clin Cancer Res.

[5] Treglia G, Annunziata S, Pizzuto DA, Giovanella L, Prior JO, Ceriani L (2019). Detection rate of (18)F-Labeled PSMA PET/CT in biochemical recurrent prostate cancer: a systematic review and a meta-analysis. Cancers (Basel).

[6] Fendler WP, Schmidt DF, Wenter V (2016). 68Ga-PSMA PET/CT detects the location and extent of primary prostate cancer. J Nucl Med.

[7] Woythal N, Arsenic R, Kempkensteffen C (2018). Immunohistochemical validation of PSMA expression measured by (68)Ga-PSMA PET/CT in primary prostate cancer. J Nucl Med.

[8] Koerber SA, Utzinger MT, Kratochwil C (2017). (68)Ga-PSMA-11 PET/CT in newly diagnosed carcinoma of the prostate: correlation of intraprostatic PSMA uptake with several clinical parameters. J Nucl Med.

[9] Zamboglou C, Drendel V, Jilg CA (2017). Comparison of (68)Ga-HBED-CC PSMA-PET/CT and multiparametric MRI for gross tumour volume detection in patients with primary prostate cancer based on slice by slice comparison with histopathology. Theranostics.

[10] Eiber M, Weirich G, Holzapfel K (2016). Simultaneous ^68^Ga-PSMA HBED-CC PET/MRI improves the localization of primary prostate cancer. Eur Urol.

[11] Donato P, Roberts MJ, Morton A (2019). Improved specificity with (68)Ga PSMA PET/CT to detect clinically significant lesions "invisible" on multiparametric MRI of the prostate: a single institution comparative analysis with radical prostatectomy histology. Eur J Nucl Med Mol Imaging.

[12] Hicks RM, Simko JP, Westphalen AC (2018). Diagnostic accuracy of (68)Ga-PSMA-11 PET/MRI compared with multiparametric MRI in the detection of prostate cancer. Radiology.

[13] Nanabala R, Anees MK, Sasikumar A, Joy A, Pillai MRA (2016). Preparation of [68 Ga]PSMA-11 for PET–CT imaging using a manual synthesis module and organic matrix based 68 Ge/ 68 Ga generator. Nucl Med Biol.

[14] Hope TA, Aggarwal R, Chee B (2017). Impact of (68)Ga-PSMA-11 PET on management in patients with biochemically recurrent prostate cancer. J Nucl Med.

[15] Afshar-Oromieh A, Malcher A, Eder M (2013). PET imaging with a 68 gallium-labelled PSMA ligand for the diagnosis of prostate cancer: biodistribution in humans and first evaluation of tumour lesions. Eur J Nucl Med Mol Imaging.

[16] Weinreb JC, Barentsz JO, Choyke PL (2016). PI-RADS prostate imaging - reporting and data system: 2015, version 2. Eur Urol.

[17] Hope TA, Goodman JZ, Allen IE, Calais J, Fendler WP, Carroll PR (2019). Metaanalysis of (68)Ga-PSMA-11 PET accuracy for the detection of prostate cancer validated by histopathology. J Nucl Med.

[18] Hirmas N, Al-Ibraheem A, Herrmann K (2019). [(68)Ga]PSMA PET/CT improves initial staging and management plan of patients with high-risk prostate cancer. Mol Imaging Biol.

[19] Hoeks CM, Barentsz JO, Hambrock T (2011). Prostate cancer: multiparametric MR imaging for detection, localization, and staging. Radiology.

[20] Park SY, Zacharias C, Harrison C (2018). Gallium 68 PSMA-11 PET/MR imaging in patients with intermediate- or high-risk prostate cancer. Radiology.

[21] Domachevsky L, Bernstine H, Goldberg N, Nidam M, Catalano OA, Groshar D (2020). Comparison between pelvic PSMA-PET/MR and whole-body PSMA-PET/CT for the initial evaluation of prostate cancer: a proof of concept study. Eur Radiol.

[22] Abd-Alazeez M, Ramachandran N, Dikaios N (2015). Multiparametric MRI for detection of radiorecurrent prostate cancer: added value of apparent diffusion coefficient maps and dynamic contrast-enhanced images. Prostate Cancer Prostatic Dis.

[23] Kranzbuhler B, Tran S, Zilli T, Burger IA (2017). 68Ga-PSMA PET/MR-positive peritoneal metastasis in the falciform ligament in recurrent prostate cancer. Clin Nucl Med.

[24] Westphalen AC, McCulloch CE, Jm Anaokar (2020). Variability of the positive predictive value of PI-RADS for prostate MRI across 26 centers: experience of the society of abdominal radiology prostate cancer disease-focused panel. Radiology.

[25] Afshar-Oromieh A, Haberkorn U, Schlemmer HP (2014). Comparison of PET/CT and PET/MRI hybrid systems using a 68Ga-labelled PSMA ligand for the diagnosis of recurrent prostate cancer: initial experience. Eur J Nucl Med Mol Imaging.

[26] Guberina N, Hetkamp P, Ruebben H (2019). Whole-body integrated [(68)Ga]PSMA-11-PET/MR imaging in patients with recurrent prostate cancer: comparison with whole-body PET/CT as the standard of reference. Mol Imaging Biol.

[27] Uslu-Besli L, Bakir B, Asa S (2019). Correlation of SUVmax and apparent diffusion coefficient values detected by Ga-68 PSMA PET/MRI in primary prostate lesions and their significance in lymph node metastasis: preliminary results of an on-going study. Mol Imaging Radionucl Ther.

[28] von Klot CJ, Merseburger AS, Boker A (2017). (68)Ga-PSMA PET/CT imaging predicting intraprostatic tumor extent, extracapsular extension and seminal vesicle invasion prior to radical prostatectomy in patients with prostate cancer. Nucl Med Mol Imaging.

[29] Burger IA, Muller J, Donati OF (2019). (68)Ga-PSMA-11 PET/MR detects local recurrence occult on mpMRI in prostate cancer patients after HIFU. J Nucl Med.

[30] Rauscher I, Duwel C, Haller B (2018). Efficacy, predictive factors, and prediction nomograms for (68)ga-labeled prostate-specific membrane antigen-ligand positron-emission tomography/computed tomography in early biochemical recurrent prostate cancer after radical prostatectomy. Eur Urol.

[31] Zacho HD, Nielsen JB, Dettmann K, Haberkorn U, Petersen LJ (2017). Incidental detection of thyroid metastases from renal cell carcinoma using ^68^Ga-PSMA PET/CT to assess prostate cancer recurrence. Clin Nucl Med.

[32] Arora S, Damle NA, Parida GK (2018). Recurrent medullary thyroid carcinoma on ^68^Ga-prostate-specific membrane antigen PET/CT: exploring new theranostic avenues. Clin Nucl Med.

[33] Roach PJ, Francis R, Emmett L (2018). The impact of (68)Ga-PSMA PET/CT on management intent in prostate cancer: results of an Australian prospective multicenter study. J Nucl Med.

[34] Calais J, Cao M, Nickols NG (2018). The utility of PET/CT in the planning of external radiation therapy for prostate cancer. J Nucl Med.

[35] Chen M, Zhang Q, Zhang C (2019). Combination of (68)Ga-PSMA PET/CT and multiparametric MRI improves the detection of clinically significant prostate cancer: a lesion-by-lesion analysis. J Nucl Med.

